# Analysis of MDM2 and TP53 genes in canine liposarcoma

**DOI:** 10.1038/s41598-024-64963-z

**Published:** 2024-06-18

**Authors:** Luisa Vera Muscatello, Dario de Biase, Thais Maloberti, Enrico di Oto, Giovanni Tallini, Valeria Pellegrino, Barbara Bacci, Paola Roccabianca, Elvio Lepri, Luca Crippa, Giancarlo Avallone

**Affiliations:** 1 Department of Veterinary Medical Sciences (DIMEVET), University di Bologna, Via Tolara di Sopra 50, 40064 Ozzano dell’Emilia, BO Italy; 2 Department of Veterinary Medicine and Animal Sciences (DIVAS), University of Milan, Lodi, Italy; 3 Department of Veterinary Medicine, University of Perugia, Perugia, Italy; 4 Istovet, Besana Brianza, Italy; 5 Department of Pharmacy and Biotechnology (FaBiT), University of Bologna, Bologna, Italy; 6 Department of Medical and Surgical Sciences (DIMEC), University of Bologna, 40138 Bologna, Italy; 7 IRCCS Azienda Ospedaliero-Universitaria di Bologna, Solid Tumor Molecular Pathology Laboratory, 40138 Bologna, Italy

**Keywords:** Genetics, Cancer, Sarcoma

## Abstract

Canine liposarcoma is an uncommon tumor that shares morphological similarities with its human counterpart. In dogs, the genetic features of this tumor are unknown and, based on immunohistochemical studies, amplification of the gene *MDM2* and the mutation of *TP53* are suspected. In this study 51 cases of primary liposarcomas were immunohistochemically stained for MDM2 and p53 and subjected to fluorescent in situ hybridization and next-generation sequencing to detect *MDM2* amplification and *TP53* mutations, respectively. MDM2 and p53 were expressed in 21 and 6 cases, respectively. *MDM2* amplification and *TP53* mutations were identified in 10 and 15 cases, respectively. Statistical analysis revealed an association of the myxoid subtype and the mitotic count with p53 expression and *TP53* mutation. No association was found between *MDM2* amplification and MDM2 expression or tumor subtype. These results suggest that despite morphological similarities, canine liposarcoma differs from its human counterpart, for which *MDM2* amplification is diagnostic for well differentiated and de-differentiated variants, and *TP53* mutations are more common in pleomorphic liposarcoma rather than the myxoid one as occur in our cases. Furthermore, canine myxoid liposarcoma likely represents a distinct disease rather than a mere morphological variant.

## Introduction

Canine liposarcoma is a relatively rare sarcoma of dogs. It predominantly originates in the subcutaneous tissue of the body’s trunk and the proximal regions of the limbs. However, there have been reports of its occurrences in other sites, such as deep soft tissue (deeper than subcutis) and the spleen^1–3^.

Canine liposarcoma can be categorized into four morphological variants that bear a striking resemblance to their human counterparts: well-differentiated, dedifferentiated, myxoid, and pleomorphic^1,2,4^. In dogs, these variants primarily serve as morphological distinctions, while in humans, they represent distinct neoplastic entities with specific genetic alterations^5^. Well-differentiated and dedifferentiated human liposarcomas are characterized by the presence of a giant ring chromosome that carries amplified copies of the *MDM2* and *CDK4* genes, which play essential roles in cell cycle regulation. These two tumor types are regarded as two ends of a morphological spectrum of the same neoplastic entity, distinct from myxoid and pleomorphic liposarcoma, with the dedifferentiated variant showing metastatic potential^5^. Human myxoid liposarcoma is known to exhibit a reciprocal translocation between chromosomes 12 and 16: t(12;16)(q13;p11), resulting in the fusion of the *DDIT3* gene with the *FUS* gene. Pleomorphic liposarcoma displays complex genetic rearrangements, and dysregulation of several tumor suppressor pathways (e.g., p53 and Rb1) is common in this subtype^5,6^.

Immunohistochemical studies have recently expanded our understanding of canine liposarcoma, revealing an overexpression of tyrosine kinase receptors (TRKs), MDM2, and p53. This suggests that TRK pathways may be involved in tumor progression, that the *MDM2* gene may be amplified as in humans, and that the *TP53 *gene could be mutated in the myxoid variant^1,7,8^. However, our knowledge regarding the genetic status of canine liposarcoma remains limited, and the few cases examined in the literature have failed to demonstrate anomalies in these genes^9^.

The aim of this study is to investigate the presence of *MDM2* gene amplification and *TP53 *gene mutations in a larger number of canine liposarcomas. We used fluorescent in situ hybridization (FISH) to assess *MDM2* amplification and next-generation sequencing (NGS) to detect *TP53 *and *MDM2 *gene mutations.

## Results

Fifty-one cases of canine liposarcoma were included in this study. Among these cases, fifteen dogs were female (4 spayed and 11 intact), thirty-two were male (1 castrated and 31 intact), and in 4 cases, sex was not reported. The age was available for 47 out of the 51 cases, ranging from 6 to 16 years, with a median age of 11 years. Among the liposarcomas, 41 affected the soft tissue, with 36 being subcutaneous, 3 intramuscular, and 2 intracavitary. Six cases were splenic, and in 4 cases, the specific site was not recorded. The distribution of subtypes included 26 well-differentiated, 10 myxoid, 8 pleomorphic, and 7 dedifferentiated cases. The mitotic count varied from 1 to 36, with a median of 5. Tumor grading classified 18 cases as grade 1, 28 as grade 2, and 5 as grade 3.

Immunohistochemistry detected the expression of MDM2 in 21 cases (including 11 well-differentiated, 5 dedifferentiated, 3 myxoid, and 2 pleomorphic cases), and the expression of p53 in 6 cases, all of which were myxoid.

### NGS

In four cases, there was an insufficient amount of tissue available in the paraffin blocks for NGS analysis, thus 47 cases were included in the study. These comprised 20 well-differentiated liposarcomas, 10 myxoid liposarcomas, 8 pleomorphic liposarcomas, and 8 dedifferentiated liposarcomas. A *TP53* mutation was identified in 15 out of the 47 liposarcomas (31.9%), which included 2 well-differentiated, 9 myxoid, 3 pleomorphic, and 1 dedifferentiated neoplasms. Out of these, ten variants were missense single nucleotide variants (SNV) mutations, and five were small indels (Table 1). Three additional variants were single nucleotide indels within a homopolymer stretch and were thus excluded from further analysis.

**Table 1:** Mutation of *TP53*identified in canine liposarcoma.

Mutation	Exon	# of cases	PolyPhen-2	Ratio PolyPhen-2
p.Pro137Val	4	2	Possiblydamaging	0.688
p.Thr244Ter	6	1	Damaging	
g.32565105insC	1	2	Damaging	
p.Ser229Tyr	6	1	Probablydamaging	1
p.S228_N235delinsILE	6	1	Damaging	
p.Val204Leu	5	1	Probablydamaging	0.999
p.Met231delT	6	1	Damaging	
p.Arg323Cys	9	1	Probablydamaging	0.994
p.Ser343Ter	9	1	Damaging	
p.Asp195Asn	5	1	Benign	0.079
p.Pro129Leu	4	1	Probablydamaging	1
p.Arg162Leu	4	1	Probablydamaging	0.999
p.Leu357Met		1	Probablydamaging	0.989
g.32565125delTC	–	1	Damaging	
p.Leu14insCfs	1	1	Damaging	

Of the 15 mutations (2 well-differentiated, 9 myxoid, 3 pleomorphic, and 1 dedifferentiated), all except one were classified as "damaging" or "possibly damaging" using the Polyphen2 tool (http://genetics.bwh.harvard.edu/pph2/). Even if PolyPhen2 was developed for the determination of variants in human beings, the analysis was conducted by performing the query with the amino acid sequence of canine reference and the corresponding positions of the variants, thus utilizing the in silico prediction of PolyPhen2. PolyPhen2 has been previously used for this type of evaluation in canine specimens in the literature^10–12^. One variant was categorized as "benign". The variant allele frequency (VAF) for these 15 mutations ranged from 8 to 76%. In two cases a nonsense mutation was detected, leading to a truncated protein at the amino acid T244 and S343; in two cases a frameshift mutation was identified (Table 1). Among these 15 variants, 5 cases were immunohistochemically positive for p53 (IHC), while 10 were negative. Interestingly, one myxoid liposarcoma that tested p53 positive by IHC showed no mutation. Notably, no missense or small indel variants were detected in the *MDM2* gene.

### FISH

Out of the total of 51 cases subjected to FISH analysis, 8 cases were found to be technically inadequate and were therefore considered indeterminate and subsequently excluded.

**Figure 1:**
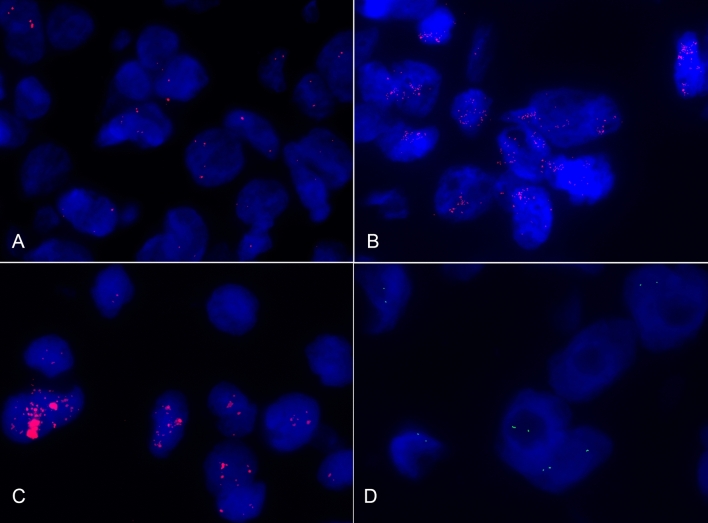
Fluorescent in situ hybridization of canine liposarcomas. (**A**) Diploid copy number of *MDM2* per nucleus (red dots). (**B**) *MDM2* amplified liposarcoma with*MDM2/GMCL1* ratio greater than 2. Increased copy number of *MDM2* with an average of 8.33 per nucleus (red dots). (**C**) *MDM2* cluster type amplification with*MDM2/GMCL1*ratio greater than 2. Closely stippled adjacent numerous red MDM2 signals that form large clusters. (**D**) Non-polysomic *MDM2* amplified liposarcoma. In green is shown the diploid *GMCL1* control gene.

Among the remaining 43 cases, ten exhibited MDMI2 amplification (23%), with *MDM2/GMCL1* ratios ranging from 2.1 to 6.5. This subset included 6 well-differentiated liposarcomas, 2 dedifferentiated, 1 myxoid, and 1 pleomorphic. The amplification pattern in 6 out of these 10 cases (60%) was identified as cluster-type (Fig. 1), characterized by closely stippled, adjacent, and numerous signals forming a large cluster within a specific area of the nucleus. In contrast, the remaining 4 cases (40%) exhibited a non-clustered amplification pattern, with signals scattered equally throughout the nucleus. Among the 10 amplified cases, 4 tested positive for MDM2 by immunohistochemistry, while 6 were negative.

Most of the cases, specifically 34 out of 43 (77%), showed no *MDM2* amplification and exhibited a diploid signal pattern. Notably, no cases displaying polysomy were observed. Among the 33 cases without *MDM2* amplification, 13 were immunohistochemically positive for MDM2, while 20 were negative.

### Statistical analysis

The occurrence of myxoid liposarcoma was significantly higher in the spleen compared to other tissues (p = 0.003), whereas the other subtypes were predominantly observed in the subcutis. Myxoid liposarcoma and dedifferentiated liposarcoma exhibited significantly higher grades in comparison to pleomorphic liposarcoma and well-differentiated liposarcoma (p = 0.0004). However, there was no significant association between the histological grade and the tumor site (p = 0.79).

A statistically significant association was found between immunohistochemical p53 positivity and *TP53* mutation (p = 0.015). However, there was no statistically significant association between MDM2 positivity and *MDM2* amplification or between MDM2 and p53 immunohistochemical expression (p = 0.92 and p = 0.38, respectively).

**Figure 2:**
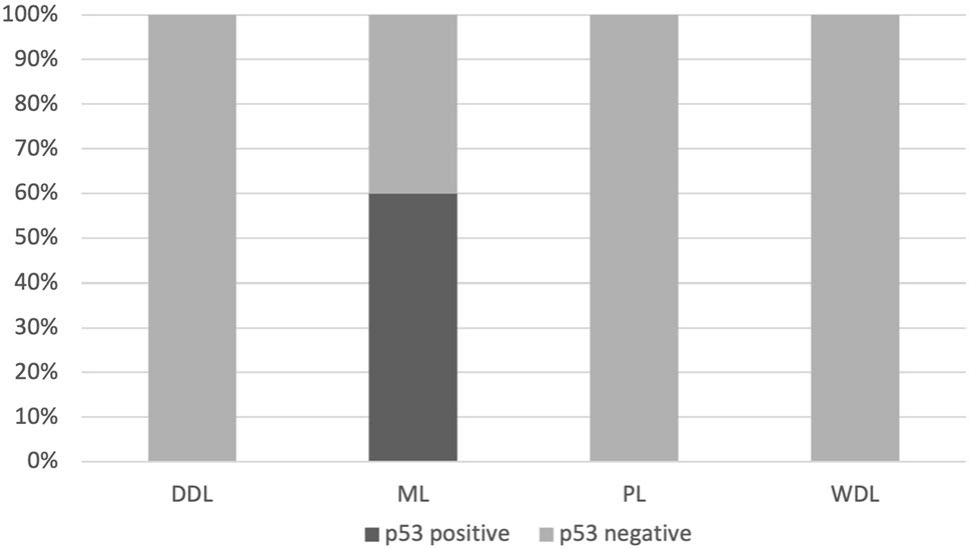
Percentage of cases immunohistochemically expressing p53 in canine liposarcoma, comparing the histotype: DDL = de-differentiated; ML = myxoid; PL = pleomorphic; WDL = well differentiated.

**Figure 3:**
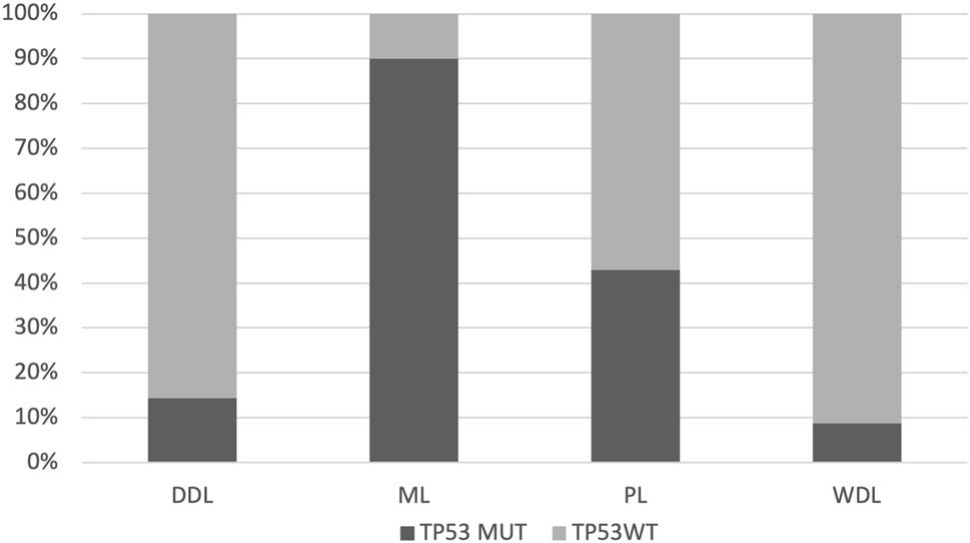
Percentage of cases harboring *TP53* mutation in canine liposarcoma, comparing the histotype: DDL = de-differentiated; ML = myxoid; PL = pleomorphic; WDL = well differentiated.

Statistical analysis revealed a strong association of the myxoid subtype (ML) with p53 immunohistochemical expression (p = 0.00003) and *TP53* mutation (p = 0.00005) (Figs. 2 and 3). No association was found between histological subtypes and MDM2 immunohistochemical expression or gene amplification (p = 0.29 and p = 0.50, respectively). Similarly, there was no association between histological grade and MDM2 anomalies as detected by IHC and FISH (p = 0.79 and p = 0.91, respectively). However, the histological grade was significantly higher in p53-positive cases (p = 0.005), but not in cases with *TP53* mutation (p = 0.18).

Mitotic count (MC) was significantly higher in myxoid (mean 16.5 ± 11.6) and dedifferentiated liposarcoma (mean 15.8 ± 11.09) in comparison to pleomorphic (mean 5.1 ± 2.03) and well differentiated liposarcoma (2.8 ± 1.67). Furthermore, MC was significantly higher in p53-positive liposarcomas (p = 0.00001) and in those with *TP53* mutation (p = 0.04).

## Discussion

In this study, we have conducted the analysis of MDM2 and TP53 genes in a case series of canine liposarcoma. The cases examined encompassed 51 cases and comprised all the histologic variants reported in dogs. This investigation revealed some notable clinical features that differ from liposarcoma in humans. It is well-established that liposarcoma is a relatively rare tumor in dogs compared to humans, where it represents one of the most frequent soft tissue sarcomas^5^. Interestingly, in humans myxoid liposarcoma is more commonly found in the lower extremities, particularly affecting the thighs while intra-abdominal cases are less common^13^. In contrast, in our dataset, half of the myxoid liposarcomas developed in the spleen, with only 2 out of 10 occurring in the soft tissue of the extremities. Furthermore, the majority of splenic liposarcomas (5 out of 6) were myxoid, suggesting that the spleen may be a preferred site for this specific sarcoma in dogs.

*TP53* mutations were prevalent in 9 out of 10 (90%) myxoid liposarcomas, while they were less frequent in other subtypes (7.7% of well-differentiated, 37.5% of pleomorphic, and 14.3% of dedifferentiated cases). Among these 9 mutations, eight were predicted to be "damaging" or "probably damaging," while one was categorized as "benign." This differs from findings in human liposarcoma, where *TP53* mutations are described in 60% of pleomorphic liposarcomas and in approximately 20% of myxoid liposarcomas^6^. Notably, a study assessing p53 immunohistochemical expression in human myxoid liposarcomas found staining predominantly in highly cellular and round-cell areas, more frequently in high-grade tumors than low-grade ones^13^. Contrarily in this caseload we did not observe a preferential p53 staining in round cell areas and a statistically significant association between p53 overexpression and grade was identified. Supporting the impact of p53 alterations on tumor biological aggressiveness is the statistically significant association of the MC with both p53 staining and *TP53* mutation. However, it should be noted that in our study, 10 out of 15 cases with a *TP53* mutation did not exhibit p53 overexpression at the immunohistochemical level. Interestingly, none of these p53-negative cases were bearing a nonsense or frameshift mutation. Therefore, this discrepancy may be attributed to reduced antibody sensitivity, although it has been shown to cross-react with the canine species^14^. The same antibody yielded similar results in a study examining p53 alterations in canine malignancies^15^ and should therefore be interpreted with caution when used to infer the mutation status of *TP53* incanine tumors. Furthermore, in one immunopositive case, a *TP53 *variant was not identified. This incongruity may also be related to an antibody-related issue or less likely, to other molecular anomalies leading to p53 stabilization within the nucleus of neoplastic cells. Interestingly, while the expression of p53 and TP53 mutation has been well-documented in canine osteosarcoma^16^, studies in canine soft tissue sarcomas are limited and have not reported associations with specific subtypes^9,17^.

The 15 mutations identified within our cohort were uniformly distributed across all the exons of the *TP53* gene. Interestingly, no variants were detected in only three exons (2, 7, 8), while in all other exons, at least one substitution was identified. This emphasizes the significance of analyzing the entire coding sequence (CDS) of the *TP53* gene and highlights that focusing solely on specific exons may result in an underestimation of potential mutations.

Immunohistochemical expression of MDM2 was identified in 21 out of 51 cases (41.17%), with the majority found in well-differentiated and dedifferentiated cases, accounting for approximately 70% of these two histotypes. This result aligns partially with the human literature, where immunohistochemistry for MDM2 is often regarded as a surrogate marker for the detection of *MDM2* gene amplification, which is diagnostic for these two liposarcoma subtypes^5^. The immunohistochemical expression of MDM2 in canine liposarcoma has been reported, and similar to what is described in people it was interpreted as suggestive of gene amplification^1^. Nevertheless, data regarding *MDM2* amplification in canine soft tissue sarcomas are scarce, and only a few cases of amplification have been demonstrated by Southern blot in rhabdomyosarcoma and malignant nerve sheath tumors, while the few cases of canine liposarcoma tested did not exhibit *MDM2* amplification^9^. In this study, *MDM2* amplification status was assessed for the first time in canine liposarcoma using FISH. *MDM2* amplification was detected in 10 out of 43 cases (23%), with the majority found in well-differentiated and dedifferentiated cases; however, 6 of these cases were negative for MDM2 immunohistochemistry. Conversely, 13 out of 33 non-amplified cases were positive at the immunohistochemistry level. These findings lead to the conclusion that neither the assessment of *MDM2* amplification by FISH nor the immunohistochemical evaluation of MDM2 protein expression can be considered specific for well-differentiated and dedifferentiated liposarcoma in dogs. These discrepancies may be attributed to sensitivity and specificity issues related to the use of probes and antibodies designed for human tissues, which, despite their cross-reactivity, may not perform optimally in canine tissues. Another hypothesis is that canine liposarcomas, despite their morphological similarities to the human counterpart, harbor a different set of genetic and proteomic alterations distinct from those of the human counterpart and that MDM2 protein expression may result, rather than from genetic amplification, from increased transcription or reduced degradation.

In summary, our findings indicate that, despite the morphological similarities between canine and its human counterpart, it appears that *MDM2* amplification is not a defining feature of canine liposarcoma, although it may occur in a minority of cases, and MDM2 protein expression could potentially contribute to its oncogenic processes. Furthermore, canine myxoid liposarcoma likely represents a distinct disease rather than a mere morphological variant, which is characterized by *TP53* mutations and a preference for involvement in the spleen.

## Materials and methods

### Case selection and histologic evaluation

Cases of histologically diagnosed canine liposarcoma were retrospectively retrieved from the archives of multiple institutions (three universities and one private diagnostic laboratory). Cases lacking sufficient paraffin-embedded tissue were excluded from the study. Subsequently, all the slides were collaboratively reviewed by two veterinary pathologists (GA and VP) using a multiheaded microscope. The diagnosis was confirmed and tumors were categorized based on histomorphology, according to the most recent classification system^4^. Mitotic count was determined as the total number of mitotic figures within 10 consecutive, non-overlapping high-power fields (HPF), corresponding to the standard area of 2.37 mm^2^ in the most cellular and proliferative regions of the tumor. The grade was determined using the grading system currently used in veterinary and human medicine^18^.

### Immunohistochemistry

Three-micrometer-thick sections underwent dewaxing and rehydration. To block endogenous peroxidase activity, they were immersed in a 3% H2O2 solution in methanol for 30 min. Subsequently, the sections were rinsed in Tris buffer (pH 7.0).

For antigen retrieval, the sections were placed in citrate buffer (pH 6.0) and heated in a microwave oven at 750 W for 2 cycles of 5 min each. Afterward, they were allowed to cool at room temperature for 20 min.

Specific antibodies for MDM2 (mouse monoclonal, clone 2A10, dilution 1:100, Abcam, Cambridge, UK) and p53 (mouse monoclonal, clone PAb 240, dilution 1:100, BD Bioscience) were applied and allowed to incubate overnight at 4 °C. Following this, the sections were incubated for 30 min at room temperature with the appropriate biotin-conjugated secondary antibody (dilution 1:200, Dako, Glostrup, Denmark).

To enhance the reaction, an avidin–biotin method (ABC kit elite, Vector, Burlingame, CA, USA) was employed, and visualization was achieved using 3,3ʹ-diaminobenzidine (0.04% for 4 min). The sections were counterstained with Harris hematoxylin, rinsed in tap water, dehydrated, and cover-slipped.

Positive controls consisted of sections from normal canine testis for MDM2 and sections from canine mammary carcinoma, where p53 expression was known, for p53. Negative controls included slides incubated with a non-specific antibody or the omission of the primary antibody.

MDM2 positivity was defined as the presence of at least one positive nucleus per high-power field (40× magnification; 0.237 mm^2^), while p53 positivity was determined when more than 5% of neoplastic cells exhibited nuclear staining, as previously documented^1,8^.

### Next generation sequencing

DNA was extracted from 2 to 4 tissue sections, each measuring 10 µm in thickness, and mounted on slides. The samples were manually scraped using a sterile blade, focusing on the area selected by the pathologist as the most representative on the hematoxylin and eosin-stained slide.

DNA amplification was carried out using an amplicon-based laboratory-developed NGS panel, enabling the amplification and sequencing of the entire Coding Sequence (CDS) of the *TP53* and *MDM2* genes (reference Canisfamiliaris 3, 48 amplicons, size 4.73 kb). *TP53* CDS and *MDM2* CDS were the only regions included in the panel. Using a targeted NGS panel has allowed to have a analytical sensitivity (10%) and costs not exceeding €300 per sample. Moreover, this panel could be easily carried into clinical practice. The median coverage obtained in this cohort using this panel was 2425×. The obtained results were analyzed using Variant Caller tool (Thermo Fisher Scientific), Integrative Genomics Viewer (IGV—v.2.12.2) and GenomeBrowse tools (https://www.goldenhelix.com/products/GenomeBrowse/index.html). Only mutations with a variant allele frequency (VAF) exceeding 5% were considered for mutational calls. Bam files of the obtained sequences were loaded on the BioPrject dataset (BioProject ID: PRJNA1118484).

### Tissue microarray

Tissue microarrays (TMA) were constructed following a previously published method^19–21^. Two cores were sampled for each case, each core measuring 3 mm in diameter. Care was taken to select areas devoid of necrosis and inflammation. Nine double-core TMA blocks were assembled, with each one containing six cases, except for the last TMA which contained three cases, and one orientation core consisting of normal hepatic parenchyma.

### Fluorescence in situ hybridization

To adapt commercially available FISH probes designed for human tissue for use with canine samples, the homology between the sequences of the canine and human *MDM2* gene was assessed using the database BLAST (Basic Local Alignment Search Tool—NCBI). The alignment revealed a 92.46% homology between the *MDM2* gene sequences of both species, thus allowing the use of human commercial probes.

Furthermore, to rule out polysomy, a suitable housekeeping gene on the same *MDM2* chromosome (CFA 10) was sought. The selection criteria included:

- No known involvement in the tumorigenesis of human and canine liposarcoma.
- Location on CFA10.
- High nucleotide sequence homology between canine and human species.

The *GMCL1* gene (germ cell-less, spermatogenesis-associated 1) met these criteria, demonstrating a 95.55% sequence homology between human and canine species according to BLAST analysis.

The tumors underwent fluorescence in situ hybridization (FISH) using a dual-core tissue microarray. The Easy FISH Pretreatment Kit (OACP IE LTD, Cork, Ireland) was utilized.

The sections were initially incubated at 75 °C for 5 min in the hybridization plate. Subsequently, the sections underwent dewaxing, dehydration, air-drying, and then incubation with a permeation solution in a water bath at 90 °C for 8 min, followed by incubation in pepsin and HCl solution at 37 °C for 19 min. The sections were then washed in a washing buffer for 5 min, and dehydration was carried out in 2-min steps using 70%, 85%, and 100% ethanol. Finally, the sections were air-dried at room temperature.

The *MDM2* gene copy number was identified using the MDM2 spectrum Orange FISH probe (catalogue number FP-054, TITAN FISH probe, OACP IE LTD, Cork, Ireland) and the Smart-ISH Solve buffer OACP IE LTD, Cork, Ireland). The GMCL1 probe, combined with its corresponding buffer (GMCL1 probe set spectrum green, catalogue number GMCL1-10-GR, Empire Genomic), was used as a control to confirm the presence of polysomy. The hybridization area was then cover-slipped and sealed with rubber cement (Supplementary Information).

The slides were incubated at 85 °C for 5 min for DNA denaturation and at 42 °C overnight for hybridization with the MDM2 probe; at 83 °C for 3 min for DNA denaturation and at 37 °C overnight for hybridization with the GMCL1 probe. Following this, the slides were washed in NP40 0.5%/2 × SSC (pH 7.0–7.5) at 75 °C for 2 min and in washing buffer for 2 min at room temperature. The slides were then dehydrated and counterstained using DAPI counterstaining solution (OACP IE LTD, Cork, Ireland).

The specificity of in-situ hybridization was further evaluated by considering the euploidy of the fibroblasts and lymphocytes adjacent to the neoplastic cells.

Evaluation of the signals' numbers and quality was made by using an Olympus BX61 fluorescent microscope equipped with relevant filters and objectives by 2 independent operators (EDO, LVM), and then images were obtained. The Cytovision Image analysis software (The CytoVision®) was employed to additionally count the number of gene copies per nucleus in the available nuclei with visible signals.

FISH assessment was conducted in accordance with the *MDM2* amplification patterns observed in human liposarcoma^22^. A *MDM2/GMCL1* ratio higher than 2 was considered indicative of *MDM2* amplification.

Samples were deemed indeterminate for *MDM2* if technical issues prevented them from being reported as either positive or negative.

### Statistical analysis

Categorical variables were reported as percentages, while for continuous variables, mean, median, standard deviation (SD), and range were provided. Fisher's test was performed to assess associations between categorical variables. One-way ANOVA was employed to determine associations between mitotic count (MC) and other categorical variables. Results were considered significant at a threshold of p ≥ 0.05.

Statistical analysis was conducted using R (version 4.2.0).

### Supplementary Information

Supplementary Information.

## Data Availability

The datasets generated and/or analysed during the current study are available in the BioProject repository, ID 1118484-BioProject-NCBI (nih.gov), accession number: PRJNA1118484.
